# The pattern of genetic diversity of different breeds of pigs based on microsatellite analysis

**DOI:** 10.18699/VJ20.669

**Published:** 2020-11

**Authors:** V.R. Kharzinova, N.A. Zinovieva

**Affiliations:** L.K. Ernst Federal Research Center for Animal Husbandry, Dubrovitsy, Moscow region, Russia; L.K. Ernst Federal Research Center for Animal Husbandry, Dubrovitsy, Moscow region, Russia

**Keywords:** pig breeds, microsatellites, genetic diversity, породы свиней, микросателлиты, генетическое разнообразие

## Abstract

One of the main tasks of genetics and animal breeding is the assessment of genetic diversity and the study of genetic relationships between different breeds and populations using molecular genetic analysis methods. We analysed the polymorphism of microsatellites and the information on the state of genetic diversity and the population structure of local breeds in Russia: the Kemerovo, the Berkshire, the Liven, the Mangalitsa, and the Civilian; in the Republic of Belarus: the Large White and the Black-and-White; and in Ukraine: the White Steppe, as well as commercial breeds of imported origin of domestic reproduction: the Large White, the Landrace, and the Duroc. The materials used for this study were the tissue and DNA samples extracted from 1,194 pigs and DNA of the UNU “Genetic material bank of domestic and wild animal species and birds” of the L.K. Ernst Federal Research Center for Animal Husbandry. Polymorphisms of 10 microsatellites (S0155, S0355, S0386, SW24, SO005, SW72, SW951, S0101, SW240, and SW857) were determined according to the previously developed technique using DNA analyser ABI3130xl. To estimate the allele pool of each population, the average number of alleles (N_A_), the effective number of alleles (N_E_ ) based on the locus, the rarified allelic richness (A_R_), the observed (H_O_ ) and expected (H_E_ ) heterozygosity, and the fixation index (F_IS_) were calculated. The degree of genetic differentiation of the breeds was assessed based on the pairwise values of F_ST_ and D. The analysis of the allelic and genetic diversity parameters of the local breeds showed that the maximum and minimum levels of polymorphism were observed in pigs of the Ukrainian White Steppe breed (N_A_ = 6.500, N_E_ = 3.709, and A_R_ = 6.020) and in pigs of the Duroc breed (N_A_ = 4.875, N_E_ = 2.119, and A_R_ = 3.821), respectively. The highest level of genetic diversity was found in the Large White breed of the Republic of Belarus (H_O_ = 0.707 and N_E_ = 0.702). The minimum level of genetic diversity was found in pigs of the imported breeds – the Landrace (H_O_ = 0.459, H_E_ = 0.400) and the Duroc (H_O_ = 0.480, H_E_ = 0.469) – indicating a high selection pressure in these breeds. Based on the results of phylogenetic analysis, the genetic origin of Large White pigs, the breeds, from which the Berkshire pigs originated, and the genetic detachment of the Landrace from the Mangalitsa breeds were revealed. The cluster analysis showed a genetic consolidation of the Black-and-White, the Berkshire, and the Mangalitsa pigs. Additionally, the imported breeds with clustering depending on the origin were characterised by a genetic structure different from that of the other breeds. The information obtained from these studies can serve as a guide for the management and breeding strategies of the pig breeds studied, to allow their better use and conservation.

## Abstract (ru)

Одной из основных задач генетики и селекции животных является оценка генетического разнообразия и исследование генетических взаимоотношений между различными породами и популяциями с помощью методов молекулярно-генетического анализа. Нами проведен анализ полиморфизма микросател- литов и получена информация о состоянии генетического разнообразия и структуры популяций локальных пород свиней, разводимых на территории России (кемеровская, беркширская, ливенская, мангалица, ци- вильская), Республики Беларусь (крупная белая, черно-пестрая), Украины (степная белая), а также коммер- ческих пород импортного происхождения отечественной репродукции (крупная белая, ландрас, дюрок). Материалом для исследований служили пробы ткани 1194 образцов свиней из биоресурсной коллекции «Банк генетического материала животных и птиц» ФГБНУ ФИЦ ВИЖ им. Л.К. Эрнста. Полиморфизм 10 STR- локусов (S0155, S0355, S0386, SW24, SO005, SW72, SW951, S0101, SW240, SW857) определяли по ранее разра- ботанной методике с помощью генетического анализатора ABI3130xl (Applied Biosystems, США). Для оценки аллелофонда каждой породы рассчитывали среднее число аллелей (N_A_) и эффективное число аллелей (N_E_ ) на локус, аллельное разнообразие (A_R_), вычисленное с применением процедуры рарификации, наблюдае- мую (H_O_) и ожидаемую (H_E_ ) гетерозиготность, индекс фиксации (F_IS_). Степень генетической дифференциации пород оценивали на основании попарных значений F_ST_ и D. Анализ параметров аллельного и генетического разнообразия локальных пород показал максимальный уровень полиморфности у свиней украинской степ- ной породы (N_A_ = 6.500, N_E_ = 3.709, A_R_ = 6.020), а минимальный – у свиней породы дюрок (4.875, 2.119 и 3.821 соответственно). Наиболее высокий уровень генетического разнообразия выявлен у свиней крупной белой породы Республики Беларусь (H_O_ = 0.707, H_E_ = 0.702). Минимальный уровень генетического разнообразия установлен у свиней импортных пород ландрас (H_O_ = 0.459, H_E_ = 0.400) и дюрок (H_O_ = 0.480, H_E_ = 0.469), что, возможно, указывает на высокое давление отбора в этих породах. По результатам филогенетического анали- за выявлена генетическая обособленность пород свиней корня крупной белой породы, в создании которых принимали участие беркширские свиньи, и отдаленность пород ландрас и мангалица. Кластерный анализ показал генетическую консолидированность свиней пород черно-пестрая, беркширская и мангалица. От- личной от других пород генетической структурой характеризовались также импортные породы свиней с кластеризацией в зависимости от происхождения. Информация, полученная в ходе исследований, может служить руководством для стратегий управления и разведения изученных пород свиней с целью лучшего их использования и сохранения.

## Introduction

Currently, the industrial production of pork is based on the use of a limited number of commercial breeds of imported pigs. These breeds are well adapted for use in intensive production systems, aimed at maximising the genetic potential of productivity (Muñoz et al., 2019). Along with breeds of imported origin, there are local breeds that are carriers of unique forms of variability and constitute the national genetic resources of agricultural animal species. Despite their small size, local breeds have not lost their importance in the modern conditions of the development of animal husbandry. Having a lower productivity compared to commercial ones, such breeds are characterised by a greater individual variability, constitutional strength, stress resistance and good adaptation to local climatic conditions (Kharzinova et al., 2017).

Nowadays, local breeds are considered irreplaceable genetic resources for the creation of geographically oriented systems for organic production of livestock products. According to Stolpovsky (2013), due to the inclusion of transnational livestock industries in the agriculture world, there is a danger of a reduction of the national genetic resources, dependence on food imports, and breeding achievements, and there is a threat of globalisation of the spread of infections and hidden genetic defects. This implies an increasing importance not only to study the gene pool of species of foreign origin of the animals but also the conservation of genetic resources of the local breeds.

According to the guidelines for the development of national plans for the management of farm animal genetic resources (FAO, 1998), FAO proposes an integrated global management of farm animal genetic resources using microsatellite reference markers (short tandem repeats, STR) (Egito et al., 2007). To date, there are many publications that show the applied importance of STR for characterising the genetic diversity and structure of pig breeds for commercial (Zinovieva et al., 2012; Vrtková et al., 2012; Szmatoła et al., 2016) and local breeding (Kaul et al., 2002; Kramarenko et al., 2018). However, comparative studies of the entire variety of local and commercial breeds of pigs bred in Russia have not yet been carried out.

The aim of this study was to characterise the genetic diversity and population structure of eight local and three commercial pig breeds based on the analysis of microsatellites.

## Materials and methods

The object of research was biological material obtained from 1,194 pigs, which was stored in the UNU “Genetic material bank of domestic and wild animal species and birds” of the L.K. Ernst Federal Research Centre for Animal Husbandry. Tissue samples (ear pinch) were used as biological material. The presented sample included eight local breeds bred in Russia: the Kemerovo (Kemerovo region, KEM, n = 35), the Berkshire (Yaroslavl region, BERK, n = 80), the Liven (Orel region, LIV, n = 67), the Mangalitsa (Altai Territory, MNG, n = 52), the Civilian (Republic of Chuvashia, CVL, n = 43); The Republic of Belarus: the Large White (BLW, n = 47) and the Black-and-White (BBP, n = 98); and Ukraine: the White Steppe (LWUK, n = 61), as well as three commercial breeds of imported origin of domestic reproduction, bred in the breeding and genetic centres of Oryol, Voronezh, and Lipetsk regions: the Large White (LW, n = 241), the Landrace (LDR, n = 250), and the Duroc (DUR, n = 223).

DNA isolation was performed using DNA-Extraction kits for genomic DNA isolation (ZAO “Syntol”, Russia), in accordance with the manufacturer’s protocol. Analysis of polymorphisms of ten microsatellites (S0155, S0355, S0386, SW24, SO005, SW72, SW951, S0101, SW240, and SW857) was carried out according to the previously described method (Kharzinova et al., 2018). The results of the amplified fragments were visualised using fragment analysis by using Gene Mapper v. 4 software (Applied Biosystems, USA).

Analysis of population genetic parameters, the degree of genetic differentiation based on matrices of pairwise values of F_ST_ and D and the construction of phylogenetic trees using the Neighbor-Net algorithm were performed in GenAlEx 6.503 (Peakall, Smouse, 2012), SplitsTree 4.14.5 software (Huson, Bryant, 2006) and R package ʻdiveRsityʼ, with subsequent visualisation using the package ‘pophelper’ (Keenan et al., 2013).

The genetic structure of the studied pig breeds was assessed using principal component analysis (PCA) in R package ‘аdegenet’ (Jombart, 2008) and was visualised using R package ‘ggplot2’ (Wickham, 2009) and through clustering using STRUCTURE 2.3.4 software (Pritchard et al., 2000), using a mixed model (the number of assumed clusters, K – from 1 to 20; length of the burn-in period – 100,000; model of Markov chains of Monte Carlo – 100,000). For each value of K, 10 iterations were performed. Structure Harvester (Earl, von Holdt, 2012) was used to determine the optimal number of clusters (ΔK), according to the method proposed by Evanno et al. (2005). Source files were generated in Microsoft Excel format and R 3.5.0 software environment (R Core Team).

## Results and discussion

In the analysis of genotypes of ten microsatellites for the entire sample, 69 alleles were detected, which exceeded the number of alleles (48 alleles) detected in the molecular genetic analysis of the Chinese pig breed with a similar number of markers (Yue, Wang, 2003). Locus SW951 had the lowest number of alleles (5 alleles). A similar trend for this locus was revealed in studies of pigs bred in Ukraine (2 alleles) (Kramarenko et al., 2018) and Thailand (7 alleles) (Charoensook et al., 2019). The greatest number of alleles (22) was found at the SO005 locus, which was consistent with the results of the studies by Guastella et al. (2010) and Šalamon et al. (2019), in which this locus exceeded the others in the number of alleles: 19 and 17 alleles, respectively. The minimum mean values of both observed (H_O_) and expected (H_E_) heterozygosity were noted at the SW951 locus: 0.437 ± 0.067 and 0.482 ± 0.071, respectively. Locus SW857 had the maximum values of indicators: H_O_ = 0.868 ± 0.018 and H_E_ = 0.783 ± 0.018.

The analysis of the distribution of genotype frequencies to the Hardy–Weinberg genetic equilibrium for the entire sample (Table 1) showed significant deviations from the state of genetic equilibrium at individual loci in all the breeds studied. In Landrace pigs, deviations from the genetic equilibrium were found at all loci, in Duroc and Large White pigs, at nine and eight loci, respectively. It should be noted that local breeds of pigs were inferior to commercial breeds in terms of the number of loci with significant deviations from the state of genetic equilibrium. The number of such loci varied from three in the Liven breed to seven in the Ukrainian White Steppe breed. These findings can indicate greater selection pressures in commercial pig breeds, compared to local breeds. Of the ten studied loci, highly significant deviations from the genetic equilibrium were established for the SO005 locus, according to Hardy–Weinberg ( p < 0.001).

**Table 1.**
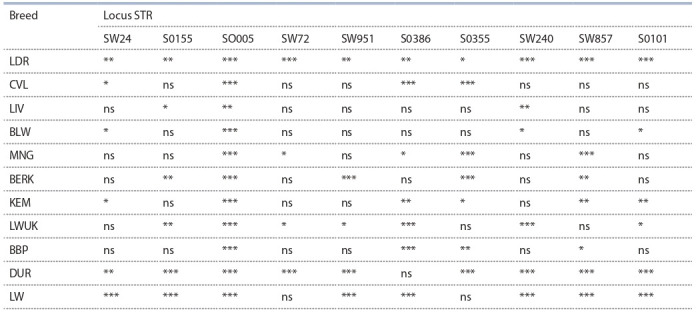
Results of the test of ten microsatellites in the analysis of the studied breeds of pigs, for compliance with the Hardy–Weinberg genetic equilibrium Note. * p < 0.05; ** p < 0.01; *** p < 0.001; ns – not significant. Breed hereinafter: KEM – the Kemerovo, BERK – the Berkshire, LIV – the Liven, MNG – the Mangalitsa, CVL – the Civilian; BLW – the Large White and BBP – the Blackand- White of The Republic of Belarus, LWUK – the White Steppe of Ukraine, LW – the Large White, LDR – the Landrace, DUR – the Duroc.

An interesting research result published by Kramarenko et al. (2018) showed that in pigs of the Duroc breed bred in the regions of Ukraine, eight out of twelve loci had insignificant deviations from the state of genetic equilibrium.

To assess the degree of genetic diversity of populations and breeds, two main indicators are most often used – the level of polymorphism and the degree of homozygosity (heterozygosity) (Khrabrova et al., 2011), whose results are presented in Table 2. The minimum values of the average number of alleles per locus (N_A_ = 4.875) were observed in three breeds: CVL, MNG, and DUR, and the maximum values (more than 6.000) were observed in Landrace pigs (LDR, N_A_ = 6.001) and in the Large White breed bred in the territories of our country (LW, N_A_ = 6.250) and in Ukraine (LWUK, N_A_ = 6.500). The values of the number of effective alleles per locus (N_E_) ranged from 2.119 (DUR) to 3.709 (LWUK).

**Table 2.**
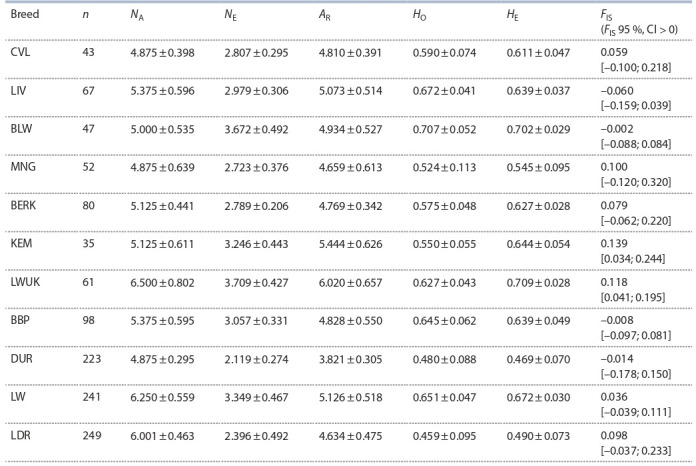
Parameters of genetic diversity of the studied breeds of pigs based on the microsatellite analysis Note. n – the number of samples; N_A_ – the average number of alleles per locus; N_E_ – the number of effective alleles per locus; A_R_ – allelic diversity; H_O_ – the observed heterozygosity; H_E_ – the expected heterozygosity; F_IS_ – inbreeding coefficient with a 95 % confidence interval.

Another measure that characterises the level of polymorphism is allelic diversity (A_R_), which is considered as a strong indicator of the evolutionary potential of a population (Allendorf, 1986; Caballero, García-Dorado, 2013), and it has been suggested that this indicator is of key importance for the conservation and management of the population (Greenbaum et al., 2014). The minimum values of this indicator, which were corrected using the rarefaction method, were detected for DUR – A_R_ = 3.821, and were maximal in LWUK – A_R_ = 6.020. According to Greenbaum et al. (2014), a decrease in allelic diversity may lead to a decrease in the population’s ability to adapt to future environmental changes. Moreover, according to Wagner (2008), there is evidence that a high allelic diversity, even of simple neutral alleles, increases the evolutionary potential by making less genotypic space available for mutational events.

To date, the most commonly used indicators of the genetic characteristics of populations presented in most studies (Vonholdt et al., 2008; Toro et al., 2009; Andras et al., 2011) are the observed (H_O_) and expected (H_E_ ) heterozygosity (Greenbaum et al., 2014). The H_O_ in the studied breeds of pigs ranged from 0.459 ± 0.095 for LDR to 0.707 ± 0.052 for BLW. According to some authors, a decrease in H_O_ can lead to a decrease in the average fitness of individuals, and, therefore, this indicator has clear ecological consequences (Reed, Frankham, 2003; Szulkin et al., 2010). Moderate levels of H_E_ (above 0.5) were observed in nine pig breeds, ranging from 0.545 ± 0.095 for MNG, to 0.709 ± 0.028 for LWUK. Pigs of the Duroc and the Lansrace breeds were an exception, in which this indicator had minimum values: 0.469 ± 0.070 and 0.490 ± 0.073, respectively.

According to the fixation index values, a slight lack of heterozygotes was found in seven breeds of pigs (CVL, MNG, BERK, KEM, LWUK, LW, and LDR) with a variation of positive values of the indicator from 0.036 for LW, to 0.139 for KEM. However, for these breeds, with the exception of KEM and LWUK, the 95 % confidence interval (CI) of the fixation index included the zero value, which indicates nonsignificant deviations in the number of heterozygotes from the theoretically expected, in these breeds. A slight shift in the genetic balance towards an excess of heterozygotes was noted in four breeds: LIV, BLW, BBP, and DUR, in which the fixation index had negative values, which amounted to 0.060, 0.002, 0.008 and 0.014, respectively.

Among the local breeds, the maximum level of polymorphism was observed in LWUK (N_A_ = 6.500, N_E_ = 3.709, A_R_ = 6.020), and the maximum level of genetic diversity was found in BLW (H_O_ = 0.707, H_E_ = 0.702). At the same time, pigs of the Mangalitsa breed had minimum values for all the analysed parameters: N_A_ = 4.875, N_E_ = 2.723, A_R_ = 0.659, H_O_ = 0.524, and H_E_ = 0.545. However, in an study by Druml et al. (2012), the values of genetic parameters characterising the level of genetic diversity of pigs of the Mangalitsa breed of Austria and the National Reserve of Serbia were even lower: N_A_ = 3.8, H_O_ = 0.49, and H_E_ = 0.54 and N_A_ = 3.94, H_O_ = 0.58, and H_E_ = 0.54, respectively. When comparing the animals of imported origin of domestic reproduction, the group of large white pigs exceeded the other two in all aspects: N_A_ = 6.250, N_E_ = 3.349, A_R_ = 5.126, H_O_ = 0.651, and H_E_ = 0.672. Of all the studied pig breeds, the minimum level of polymorphism and genetic diversity was found in the Duroc breed: N_A_ = 4.875, N_E_ = 2.119, A_R_ = 3.821, H_O_ = 0.480, and H_E_ = 0.469. A similar trend towards a relatively low level of genetic diversity of this breed was noted in the works of other authors. In a comparative analysis of local breeds of Brazil with pigs of specialised breeds (Duroc, Landrace, and Large White), the minimum values of both the average number of alleles per locus and the effective number of alleles were identified in the Duroc breed, which amounted to N_A_ = 3.65 and N_E_ = 3.01 (da Silva et al., 2011). In his work, Szmatoła et al. (2016), while studying the genetic diversity of four commercial breeds and one local breed of pigs in Poland using five microsatellites, he revealed the lowest values of the average number of alleles per locus (N_A_ = 4.6), the number of effective alleles per locus (N_E_ = 2.78) and allelic diversity (A_R_ = 4.6). However, the studies by Kim et al. (2005), which focused on the description of the genetic diversity and population structure of four European, two Korean and three Chinese pig breeds, showed that Duroc pigs outnumbered others in these parameters. At the same time, local Korean pigs showed consistently low levels of allelic diversity and heterozygosity, while Chinese pig breeds, except for the Wuzhishan breed, had a relatively high degree of genetic diversity compared to commercial and local Korean pig breeds. The lower values of population genetic parameters detected in our work, both in pigs of the Mangalitsa and the Duroc breeds, possibly indicates a high selection pressure and a minimal or no migration of new genes in the breeds.

**Fig. 1.**
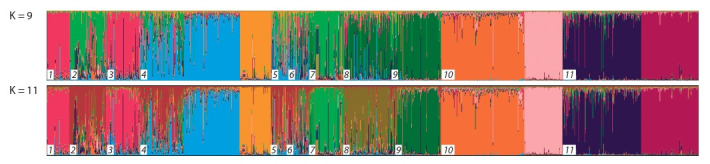
Results of the cluster analysis of eleven pig breeds based on microsatellites using the STRUCTURE 2.3.4 software. Breeds: 1 – Civilian; 2 – Liven; 3 – White Steppe of Ukraine; 4 – Large White; 5 – Large White of The Republic of Belarus; 6 – Kemerovo; 7 – Mangalitsa; 8 – Blackand- White of The Republic of Belarus; 9 – Berkshire; 10 – Duroc; 11 – Landrace.

**Fig. 2.**
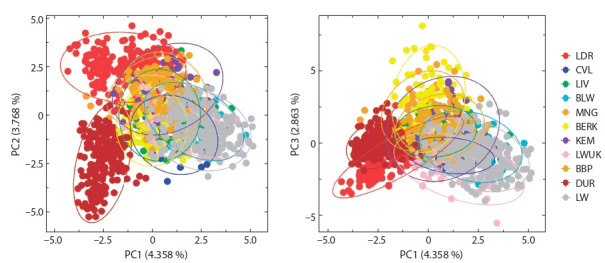
Projection of the studied samples of the pig breeds on the plane of two coordinates according to PCA analysis.

To assess the genetic structure of the studied pig breeds, Bayesian cluster analysis was carried out using STRUCTURE (Fig. 1), as well as coordination analysis, using PCA (Fig. 2). Despite the fact that the algorithm based on the values of ΔK (Earl et al., 2012) revealed that the optimal number of clusters for this sample is equal to 9, K = 9 (ΔK = 136.79), the results at K = 11 were also presented.

The breeds LIV, BLW, and LW are characterised by a mixed genetic origin. In addition, a similar genetic pattern was observed in the CVL and LWUK breeds. A clear genetic structure has been identified in black-and-white, Berkshire, and Mangalitsa pigs. The formation of several clusters of breeds of imported origin is explained by both different origins and different strategies of selection and the breeding work used in the enterprises.

Principal component analysis, the key feature that enables the projection of samples onto orthogonal coordination axes, each of which consisting of a linear combination of allelic or genotypic values (Patterson et al., 2006; Novembre et al., 2008), revealed a genetic mixing and enabled the visualisation of a slight differentiation for most of the studied breeds. Independent cluster formed by representatives of commercial breeds (Duroc, Landrace, and Large White); at the same time, local breeds formed overlapping arrays. According to Jolliffe and Cadima (2016), the lack of clear clustering does not mean the absence of differences but may indicate the similarity of the largest source of variability. In addition, this analysis made it possible to characterise the range of variability in three components. The first component was responsible for most of the genetic variability of the entire data set (4.3 %), while the second and third components reflected 3.7 and 2.8 % of genetic variability, respectively.

To assess the degree of differentiation of populations, two main classes of indicators that determine the quantitative structure of populations are used: fixation indices F_ST_ and Nei’s G_ST_, and indicators of allelic differentiation, such as Jost’s D and differential entropy (Jost et al., 2018). One of the most commonly used indicators in population genetic studies is the standard method for estimating the F_ST_ fixation index, described by Weir and Cockerham (1984). However, when calculating the genetic distances based on the variability of highly polymorphic markers, the values of the indicator may be shifted (Meirmans, Hedrick, 2011; Hopper et al., 2018). Therefore, we additionally performed calculations of the D index proposed by Jost (2008) that takes into account the proportion of allelic variations in populations (Table 3).

**Table 3.**
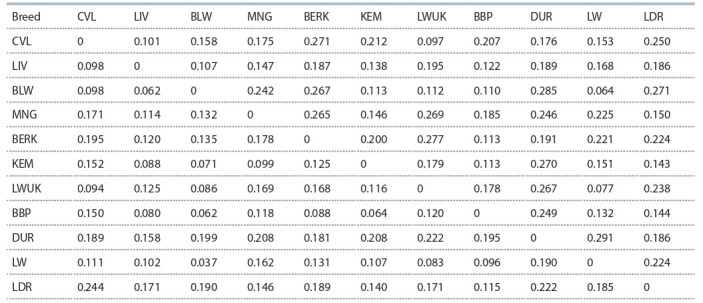
Genetic distances between the studied breeds of pigs based on the microsatellite analysis Note. D values are shown above the diagonal. The F_ST_ values are below the diagonal for pairwise comparison.

The greatest genetic affinity for both indicators was found for pigs of the Russian and Belarusian populations of the Large White breed: LW/BLW F_ST_ = 0.037, D = 0.064. However, regarding the maximum values of the indices, differences were detected: the greatest genetic distance, according to the F_ST_ fixation index, is characteristic of the LDR/CVL = 0.244 group, and the LW/DUR = 0.291 group, according to the D index.

**Fig. 3.**
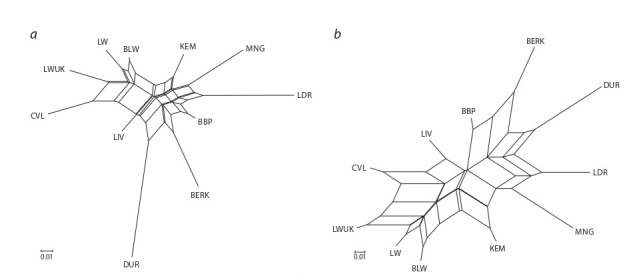
Phylogenetic dendrogram of the genetic relationships of the studied pig breeds based on the pairwise genetic distances matrix F_ST_ (a) and Jost’s D (b) using the Neighbor-Net algorithm.

To visualise the genetic degree of closeness of the studied pig breeds, the numerical matrices of the pairwise genetic distances, F_ST_ and D, were visualised using the Neighbor-Net algorithm and are presented in Figure 3. A separate massif was formed by groups of root pigs of a large white breed (CVL, LWUK, LW, and BLW) and adjacent branches of the Kemerovo and Liven breeds. A separate branch is a cluster of breeds in the creation of which pigs with the blood of the Berkshire breed took part: BBP, BERK, and DUR. The Landrace and the Mangalitsa animals are presented in a separate cluster.

## Conclusion

Our studies were aimed at analysing the genetic diversity and studying the relationship between eight local pig breeds and three pig breeds of imported origin of domestic reproduction. In general, the studied local breeds exceeded the groups of imported pigs in both allelic and genetic diversity, which is probably explained by the lack of a practical program of continuous improvement of specific characteristics, to which commercial breeds are subject. On the contrary, the maximum positive values of the fixation index were detected in local breeds (Kemerovo and Ukrainian White Steppe), which can lead to a shift in the genetic equilibrium towards a lack of heterozygotes. The analysis of the main components, carried out on the basis of the allele frequencies of the studied breeds of pigs, made it possible to characterise the range of variability and trace the main patterns of population genetic differentiation of individuals of the studied breeds of pigs. The information obtained from these studies can guide the management and breeding strategies of the studied breeds in order to better use and conserve them. At the same time, further studies of pigs, both local and specialised breeds, with many microsatellites, using mitochondrial DNA and single nucleotide polymorphism analysis, seem necessary. Future genetic progress will mainly depend on the availability of sufficient genetic variation, and a more holistic understanding of the state of genetic diversity and the structure of pig breeds will bring tremendous benefits for the entire pig industry.

## Conflict of interest

The authors declare no conflict of interest.

## References

Allendorf F.W. Genetic drift and the loss of alleles versus heterozygosity.
Zoo. Biol. 1986;5:181-190.

Andras J., Kirk N., Harvell C. Range-wide population genetic structure
of Symbiodinium associated with the Caribbean sea fan coral, Gorgonia
ventalina. Mol. Ecol. 2011;20:2525-2542. DOI 10.1111/j.1365-
294X.2011.05115.x.

Caballero A., García-Dorado A. Allelic diversity and its implications
for the rate of adaptation. Genetics. 2013;195(4):1373-1384. DOI
10.1534/genetics.113.158410.

Charoensook R., Gatphayak K., Brenig B., Knorr C. Genetic diversity
analysis of Thai indigenous pig population using microsatellite
markers. Asian-Australas. J. Anim. Sci. 2019;32(10):1491-1500.
DOI 10.5713/ajas.18.0832.

da Silva E.C., Dutra W.M., Jr., Ianella P., Filho M.A.G. de Oliveira
C.J.P., de Moura Ferreira D.N., Caetano A.R., Paiva S.R. Patterns
of genetic diversity of local pig populations in the State of Pernambuco.
R. Braz. Zootec. 2011;40(8):1691-1699. DOI 10.1590/S1516-
35982011000800010.

Druml T., Salajpal K., Dikic M. Genetic diversity, population structure
and subdivision of local Balkan pig breeds in Austria, Croatia, Serbia
and Bosnia-Herzegovina and its practical value in conservation
programs. Genet. Sel. Evol. 2012;44:5. DOI 10.1186/1297-9686-44-5.

Earl D.A., von Holdt B.M. Structure Harvester: a website and program
for visualizing Structure output and implementing the Evanno
method.
Conserv. Genet. Resour. 2012;4:359-361. DOI 10.1007/
s12686-011-9548-7.

Egito A.A., Paiva S.R., Albuquerque M.S., Mariante A.S., Almeida
L.D., Castro S.R., Grattapaglia D. Microsatellite based genetic
diversity and relationships among ten Creole and commercial cattle
breeds raised in Brazil. BMC Genet. 2007;8:83-97. DOI 10.1186/
1471-2156-8-83.

Evanno G., Regnaut S., Goudet J. Detecting the number of clusters of individuals
using the software STRUCTURE: a simulation study. Mol.
Ecol. 2005;14:2611-2620. DOI 10.1111/j.1365-294X.2005.02553.x.

FAO: Measurement of Domestic Animal Diversity (MoDAD): Original
Working Group Report. Rome, FAO, 1998.

Greenbaum G., Templeton A.R., Zarmi Y., Bar-David S. Allelic richness
following population founding events – a stochastic modeling
framework incorporating gene flow and genetic drift. PLoS One.
2014;10(3):e0119663.

Guastella A.M., Criscione A., Marletta D., Zuccaro A., Chie L., Bordonaro
S. Molecular characterization and genetic structure of the
Nero Siciliano pig breed. Genet. Mol. Biol. 2010;33(4):650-656.
DOI 10.1590/S1415-47572010005000075.

Hopper J.V., McCue K.F., Pratt P.D., Duchesne P., Grosholz E.D.,
Hufbauer R.A. Into the weeds: matching importation history to
genetic consequences and pathways in two widely used biological
control agents. Evol. Appl. 2018;12(4):1-18. DOI 10.1111/eva.
12755.

Huson D.H., Bryant D. Application of phylogenetic networks in evolutionary
studies. Mol. Biol. Evol. 2006;23(2):254-267. DOI 10.1093/
molbev/msj030.

Jolliffe I.T., Cadima J. Principal component analysis: a review and
recent developments. Philos. Trans. R. Soc. A. 2016;374(2065):
20150202. DOI 10.1098/rsta.2015.0202.

Jombart T. adegenet: a R package for the multivariate analysis of genetic
markers. Bioinformatics. 2008;24:1403-1405. DOI 10.1093/
bioinformatics/btn129.

Jost L. GST and its relatives do not measure differentiation. Mol. Ecol.
2008;17:4015-4026. DOI 10.1111/j.1365-294X.2008.03887.x.

Jost L., Archer F., Flanagan S., Gaggiotti O., Hoban S., Latch E. Differentiation
measures for conservation genetics. Evol. Appl. 2018;
11(7):1139-1148. DOI 10.1111/eva.12590.

Kaul R., Singh A., Vijh R.K., Tantia M.S., Beh R. Evaluation of the
genetic variability of 13 microsatellite markers in native Indian pigs.
J. Genet. 2002;80:149-153. DOI 10.1007/BF02717911.

Keenan K., McGinnity P., Cross T.F., Crozier W.W., Prodöhl P.A.
diveRsity:
an R package for the estimation and exploration of population
genetics parameters and their associated errors. Methods Ecol.
Evol. 2013;4:782-788. DOI 10.1111/2041-210X.12067.

Kharzinova V.R., Karpushkina T.V., Deniskova T.E., Kostyunina O.V.,
Zinovieva N.A. Populational-genetic characterization of White
Large, Landrace, and Duroc pig breeds using microsatellites. Zootekhniya
= Zootechnics. 2018;4:2-7. (in Russian)

Kharzinova V.R., Kostyunina O.V., Zinovieva N.A. Сomparative characterization
of the allele pool of local pig breeds based on microsatellite analysis. Svinovodstvo = Pig Breeding. 2017;1:5-7. (in Russian)

Khrabrova L.A., Kalinkova L.V., Zaitseva M.A. Guidelines for the Use
of Horse DNA Analysis for the Assessment of Genetic Resources
in
Horse Breeding. Divovo, 2011. (in Russian)

Kim T.H., Kim K.S., Choi B.H., Yoon D.H., Jang G.W., Lee K.T.,
Chung H.Y., Lee H.Y., Park H.S., Lee J.W. Genetic structure of
pig breeds from Korea and China using microsatellite loci analysis.
J. Anim. Sci. 2005;83:2255-2263.

Kramarenko S.S., Lugovoy S.I., Kharzinova V.R., Lykhach V.Y., Kramarenko
A.S., Lykhach A.V. Genetic diversity of Ukrainian local pig
breeds based on microsatellite markers. Regul. Mech. Biosyst. 2018;
9(2):177-182. DOI 10.15421/021826.

Meirmans P.G., Hedrick P.W. Assessing population structure: F(ST)
and related measures. Mol. Ecol. Resour. 2011;11(1):5-18. DOI
10.1111/j.1755-0998.2010.02927.x.

Muñoz M., Bozzi R., García-Casco J., Núñez Y., Ribani A., Franci O.,
García F., Škrlep M., Schiavo G., Bovo S., Utzeri V.J., Charneca
R., Martins J.M., Quintanilla R., Tibau J., Margeta V., Djurkin-
Kušec I., Mercat M.J., Riquet J., Estellé J., Zimmer C., Razmaite V.,
Araujo J.P., Radović Č., Savić R., Karolyi D., Gallo M., Čandek-Potokar
M., Fernández A.I., Fontanesi L., Óvilo C. Genomic diversity,
linkage disequilibrium and selection signatures in European local
pig breeds assessed with a high density SNP chip. Sci. Rep. 2019;9:
13546. DOI 10.1038/s41598-019-49830-6.

Novembre J., Johnson T., Bryc K., Kutalik Z., Boyko A.R. Genes mirror
geography within Europe. Nature. 2008;456:98-101.

Patterson N., Price A.L., Reich D. Population structure and Eigen
analysis. PLoS Genet. 2006;2(12):e190. DOI 10.1371/journal.pgen.
0020190.

Peakall R., Smouse P.E. GenAlEx 6.5: genetic analysis in Excel.
Population genetic software for teaching and research – an update.
Bioinformatics. 2012;28:2537-2539. DOI 10.1093/bioinformatics/
bts460.

Pritchard J.K., Stephens M., Donnelly P. Inference of population structure
using multilocus genotype data. Genetics. 2000;155:945-959.
pmid: 10835412.

R Core Team. R: a language and environment for statistical computing.
R Foundation for statistical computing. Vienna, Austria, 2012.
Available at http://www.Rproject.org

Reed D.H., Frankham R. Correlation between fitness and genetic diversity.
Biol. Conserv. 2003;17:230-237.

Šalamon D., Margeta P., Klišanić V., Menčik S., Karolyi D., Mahnet Ž.,
Škorput D., Luković Z., Salajpal K. Genetic diversity of the Banija
spotted pig breed using microsatellite markers. J. Centr. Eur. Agric.
2019;20:36-42.

Stolpovsky Yu.A. Population genetics studies underlying preservation
of domesticated animal species gene pools. Vavilovskii Zhurnal
Genetiki i Selektsii = Vavilov Journal of Genetics and Breeding.
2013;17(4/2):900-915. (in Russian)

Szmatoła T., Ropka-Molik K., Tyra M., Piórkowska K., Żukowski K.,
Oczkowicz M., Blicharski T. The genetic structure of five pig breeds
maintained in Poland. Ann. Anim. Sci. 2016;16(4):1019-1027. DOI
10.1515/aoas-2016-0006.

Szulkin M., Bierne N., David P. Heterozygosity-fitness correlations:
a time for reappraisal. Evolution. 2010;64:1202-1217.

Toro M., Fernández J., Caballero A. Molecular characterization of
breeds and its use in conservation. Livest Sci. 2009;120:174-195.

Vonholdt B.M., Stahler D.R., Smith D.W., Earl D.A., Pollinger J.P. The
genealogy and genetic viability of reintroduced Yellowstone grey
wolves. Mol. Ecol. 2008;17:252-274.

Vrtková I., Stehlík L., Putnová L., Kratochvílová L., Falková L. Genetic
structure in three breeds of pigs populations using microsatellite
markers in the Czech Republic. Research in Pig Breeding. 2012;
6(2):83-87.

Wagner A. Robustness and evaluability: a paradox resolved. Proc. Biol.
Sci. 2008;275:91-100.

Weir B.S., Cockerham C.C. Estimating F-Statistics for the analysis
of population structure. Evolution. 1984;38(6):1358-1370. DOI
10.2307/2408641.

Wickham H. ggplot2: Elegant graphics for data analysis. NY: Springer-
Verlag, 2009.

Yue G.H., Wang G.L. Molecular genetic analysis of the Chinese Erhualian
pig breed. S. Afr. J. Anim. Sci. 2003;33(3):159-165.

Zinovieva N.A., Kharzinova V.R., Sizareva E.I., Gladyr’ E.A.,
Kostyunina O.V., Lugovoi S.I., Tapiha V.A., Gamko L.N., Ovseenko
E.V., Shavyrina K.M., Ernst L.K. Evaluation of the contribution
of different pig populations to the genetic diversity of the large white
breed. Selskokhozyaystvennaya Biologiya = Agricultural Biology.
2012;6:35-42. (in Russian)

